# PPARγ Agonist Pioglitazone Prevents Hypoxia-induced Cardiac Dysfunction by Reprogramming Glucose Metabolism

**DOI:** 10.7150/ijbs.98387

**Published:** 2024-08-06

**Authors:** Yijin Wang, Ru Zhang, Qian Chen, Zhangwen Lei, Caiyu Shi, Yifei Pang, Shan'an Zhang, Linjie He, Longtao Xu, Jinliang Xing, Haitao Guo

**Affiliations:** 1 College of Life Sciences, Northwest University, Xi'an, 710069, China.; 2 The Key Laboratory of Aerospace Medicine, Ministry of Education, Fourth Military Medical University, 710069, China.; 3 School of Medicine, Northwest University, Xi'an, 710069, China.; 4 College of Basic Medicine, Fourth Military Medical University, Xi'an, 710032, China.; 5 State Key Laboratory of Cancer Biology and Department of Physiology and Pathophysiology, Fourth Military Medical University, Xi'an, 710032, China.

**Keywords:** Hypoxia, Cardiac dysfunction, Pioglitazone, Glucose metabolic reprogramming, PPARγ, HIF-1α

## Abstract

The heart relies on various defense mechanisms, including metabolic plasticity, to maintain its normal structure and function under high-altitude hypoxia. Pioglitazone, a peroxisome proliferator-activated receptor γ (PPARγ), sensitizes insulin, which in turn regulates blood glucose levels. However, its preventive effects against hypoxia-induced cardiac dysfunction at high altitudes have not been reported. In this study, pioglitazone effectively prevented cardiac dysfunction in hypoxic mice for 4 weeks, independent of its effects on insulin sensitivity. *In vitro* experiments demonstrated that pioglitazone enhanced the contractility of primary cardiomyocytes and reduced the risk of QT interval prolongation under hypoxic conditions. Additionally, pioglitazone promoted cardiac glucose metabolic reprogramming by increasing glycolytic capacity; enhancing glucose oxidation, electron transfer, and oxidative phosphorylation processes; and reducing mitochondrial reactive ROS production, which ultimately maintained mitochondrial membrane potential and ATP production in cardiomyocytes under hypoxic conditions. Notably, as a PPARγ agonist, pioglitazone promoted hypoxia-inducible factor 1α (HIF-1α) expression in hypoxic myocardium. Moreover, KC7F2, a HIF-1α inhibitor, disrupted the reprogramming of cardiac glucose metabolism and reduced cardiac function in pioglitazone-treated mice under hypoxic conditions. In conclusion, pioglitazone effectively prevented high-altitude hypoxia-induced cardiac dysfunction by reprogramming cardiac glucose metabolism.

## Introduction

Hypoxia, resulting from decreased in barometric pressure, is the most challenging aspect of high-altitude environments^1^. Globally, more than 40 million people reside in high-altitude regions and experience chronic hypoxia^2^. High-altitude natives, whether occasional or permanent dwellers in mountainous regions, can develop chronic mountain sickness, including pulmonary hypertension (PH) and heart failure (HF), despite genetic adaptations to hypoxia found in certain Tibetan and Ethiopian populations^3^-^5^. The emergence of cardiopulmonary disease is a significant indicator of maladaptation to chronic hypoxia^6^.

Several interventions have been developed to prevent high-altitude illnesses. Although oxygen supplementation through a nasal cannula or mask is a safe and effective option, its widespread use is hindered by logistic constraints and high costs^7^. Pharmacological interventions, such as acetazolamide and sildenafil, have been effective in preventing specific high-altitude illnesses. Acetazolamide prevents acute mountain sickness by promoting bicarbonate diuresis and compensating respiratory alkalosis^8^,^9^. However, it can cause side effects, such as increased diuresis and altered taste^8^,^9^. Moreover, its effectiveness in preventing chronic high-altitude illnesses remains uncertain. Sildenafil, a phosphodiesterase type-5 (PDE-5) inhibitor, induces vasodilation by inhibiting the hydrolytic breakdown of the vasodilator cGMP, effectively alleviating high-altitude pulmonary arterial hypertension^10^. Nevertheless, Liu et al. found no significant improvement in cardiac function, exercise capacity, or any measure of clinical status despite administering sildenafil to patients with heart failure with preserved ejection fraction (HFpEF)^11^. Given the limitations of current pharmacological prevention strategies, an urgent need exists to develop novel therapeutic interventions to prevent chronic cardiovascular diseases at high altitudes.

High-altitude environments induce myocardial hypoxia, leading to changes in the metabolic pathways of cardiomyocytes. Therefore, metabolic plasticity under hypoxic conditions is important for maintaining the normal structure and function of high-energy-demanding organs, such as the heart^12^. The Sherpa people have developed adaptive mechanisms to survive high-altitude hypoxia. Metabolic reprogramming is achieved by reducing activities of oxygen-consuming processes and increasing the utilization of oxygen-efficient fuel substrates, such as carbohydrates, constituting a significant defense mechanism^13^,^14^. Therefore, pharmacological interventions targeting cardiac metabolism may effectively prevent high-altitude hypoxia-induced myocardial disease.

Pioglitazone is currently the thiazolidinedione widely used to treat type 2 diabetes mellitus (T2DM). It improves hyperglycemia by reducing insulin resistance by pancreatic beta cells^15^. Pioglitazone minimizes the risk of myocardial infarction and stroke in patients with insulin resistance^16^. Additionally, a previous study indicated that treatment with pioglitazone improved left ventricular (LV) diastolic and systolic functions in patients with T2DM^17^. These findings suggest that pioglitazone has the potential to regulate metabolic reprogramming and preserve cardiac function. Notably in the Prospective Pioglitazone Clinical Trial in Macrovascular Events studies, pioglitazone did not improve mortality in patients with T2DM and severe HF^18^. However, pioglitazone does not negatively affect the function of a healthy heart^19^, suggesting that it may be an effective preventive medication for reducing the risk of myocardial disease during high-altitude hypoxia.

This study aimed to determine whether pioglitazone can prevent hypoxia-induced cardiac dysfunction at high altitudes and explore the underlying mechanisms.

## Methods

### Animals and reagents

Male C57BL/6J mice were randomly assigned to either the hypoxic or normoxic group (*n* = 6-7 per group). Mice in the hypoxic group were exposed to simulated high-altitude conditions at 5,500 m for 4 weeks by housing them in a custom-made decompression chamber (Tow-Int, Shanghai, China) to induce hypoxia by reducing atmospheric pressure. The mice in the normoxic group were housed under ambient air conditions for the same duration as those in the experimental group. During this period, mice in the hypoxic group were treated with pioglitazone via intragastric administration at a daily dose of 10 or 20mg/kg for 4 weeks. Additionally, mice were injected intraperitoneally with KC7F2, a HIF-1α inhibitor at a dose of 10mg/kg or GW9662, a PPARγ antagonist at a dose of 1mg/kg for the same duration. Pioglitazone (Sigma-Aldrich, St. Louis, Missouri, USA), KC7F2 (Selleck Chemicals, Texas, USA) and GW9662 (MedChem Express, New Jersey, USA) were dissolved in dimethyl sulfoxide (DMSO) (Sigma-Aldrich, St. Louis, Missouri, USA) diluted with saline (final concentration, 5%). Control mice were administered saline-diluted DMSO exclusively. All mice were kept under a 12-h light-dark cycle and were provided food ad libitum. All animal experiments were conducted in accordance with the guidelines of Institutional Animal Care, and the study was approved by the Fourth Military Medical University Committee on Animal Care.

### Echocardiography

Echocardiography was conducted using a Vevo 3100 echocardiography system (Fujifilm Visualsonics Inc., Toronto, ON, Canada), as previously described^20^. Mice were anesthetized with 2.5-3.0% (v/v) isoflurane mixed with air. The mice were evaluated using a toe pinch to ensure complete anesthesia. Then, the heart rate of mice was maintained at 425 ± 50 beats per minute by adjusting the concentration of isoflurane^21^. The LV ejection fraction (LVEF), LV fractional shortening (LVFS), LV systolic and diastolic internal dimensions (LVIDs/d), right ventricular (RV) tricuspid annular plane systolic excursion (TAPSE), and RV wall thickness (RVWT) were obtained in M-mode. Pulmonary artery acceleration time (PAT) and ejection time (ET) were assessed using the pulsed doppler mode.

### Measurement of right ventricular systolic pressure and RV hypertrophy

Mice were anesthetized using a 1% intraperitoneal injection of pentobarbital sodium. A 23-gauge needle connected to a pressure transducer (ADInstruments, St. Paul, MN, USA) was inserted into the RV through the diaphragm to acquire RV systolic pressure (RVSP) readings. The PowerLab data acquisition system was used to record the RVSP measurements. Following hemodynamic measurements, the hearts and lungs of the mice were flushed with a saline solution via the pulmonary artery and subsequently harvested for further analyses. RV hypertrophy was determined by calculating the weight ratio of the RV to LV plus the septum (RV/LV+Septum).

### Histological analyses

After harvesting, the heart tissues and left lungs of the mice were fixed overnight at 4 °C in a 4% paraformaldehyde solution with a pH of 7.0. Subsequently, they were embedded in paraffin and sectioned for further analysis. To determine the cross-sectional area of cardiomyocytes, FITC-labeled wheat germ agglutinin (WGA) staining was performed according to the manufacturer's instructions (Sigma-AldrichSt. Louis, Missouri, USA). Myocardial interstitial fibrosis was measured using Masson's trichrome staining kit (Servicebio, Wuhan, China) in accordance with the manufacturer's instructions. ImageJ software (NIH, Bethesda, MD, USA) was used to analyze the fibrotic area and cardiomyocyte size in the heart.

### Western blotting and quantitative real-time-polymerase chain reaction (qRT-PCR)

Mice hearts were homogenized in RIPA buffer supplemented with complete protease inhibitors, phosphorylase inhibitors and phenylmethylsulfonyl fluoride (Roche, Basle, Switzerland). The protein concentration was determined using a bicinchoninic acid kit (Beyotime, Shanghai, China). Western blotting was performed as previously described^22^. The protein bands were visualized using a chemiluminescence system (Amersham Bioscience, Buckinghamshire, UK) and quantified using the ImageJ software. The primary antibodies and working concentrations were shown in Supplementary Table 1. Total RNA was extracted from the LV of mice using the TRIzol reagent (Tiangen, Beijing, China), as previously described^23^. The extracted RNA (500 ng) was reverse transcribed into complementary DNA using the PrimeScript RT Reagent Kit (TaKaRa, Otsu, Japan). For qRT-PCR detection and analysis, the real-time was performed with TB Green (TaKaRa, Otsu, Japan) and CFX96 real-time PCR detection system (Bio-Rad, California, USA). The ΔΔCT method was used to determine the relative expression level of the gene, with actin as the internal control gene. The primer sequences are listed in Supplementary Table 2.

### Glucose and insulin tolerance tests

Glucose tolerance tests (GTTs) and insulin tolerance tests (ITTs) were conducted on awake mice after a 6-h fasting (09:00-15:00), as previously described^24^. Mice exposed to 4 weeks of hypoxia were administered an intraperitoneal injection of 2 g/kg glucose (Sigma - Aldrich, St. Louis, Missouri, USA) or 0.75 U/kg insulin (Novo Nordisk, Copenhagen, Denmark) to induce GTTs or ITTs, respectively. The glucose levels were measured using a glucometer (Roche, Basel, Switzerland). Baseline glucose levels were subtracted from subsequent measurements to calculate the baseline-corrected area under the curve (AUC) for each mouse in GTTs. AUC values higher than baseline glucose levels indicated glucose resistance. For the ITTs, the baseline-corrected inverse AUC was calculated by subtracting the baseline glucose levels and inverting the values. Lower inverse AUC values indicated reduced insulin sensitivity.

### Determination of blood glucose and plasma insulin level

Blood samples were collected from the tail vein after overnight fasting and blood glucose level was measured using a glucometer. Plasma insulin levels were determined using a mouse insulin enzyme-linked immunosorbent assay kit (Elabscience, Wuhan, China).

### Mitochondrial complex activity assay

The commercial assay kits (Solarbio, Beijing, China) were utilized to measure the activities of mitochondrial complexes I, II, III, IV and V in LV tissues according to the manufacturer's instructions. Briefly, fresh LV tissues were first homogenized in extract solution provided by assay kits and centrifuged at 4℃ for supernatant collection. Then, enzymatic reaction was carried out by following the kit's protocol. Finally, the activities of mitochondrial complexes were determined by measuring the OD values using a microplate reader (BioTek, New York, USA) at specified wavelength.

### Culturing and pioglitazone treatment of neonatal rat cardiomyocytes

Neonatal rat cardiomyocytes were isolated from rats aged 0-2 d and cultured according to a previously described protocol^25^. Briefly, the hearts of the neonatal rats were promptly excised, minced, and dissociated using 1% collagenase I at 37℃. The resulting dispersed cardiomyocytes were cultured in fresh DMEM supplemented with 10% fetal bovine serum. Subsequently, the cardiomyocytes treated with 10 μmol pioglitazone and control cardiomyocytes were cultured at 37℃ under physiological normoxia (5% O_2_) or hypoxia (1% O_2_).

### Measurement of cardiomyocyte contractility and extracellular field potential

Neonatal cardiomyocytes (4×10^4^-6×10^4^cells/well) were seeded in an NSP-96 plate and cultured in DMEM for 48 h. Cardiomyocyte contractility and coordinated ion channel activity were then assessed by monitoring impedance (IMP) and extracellular field potential (EFP) using a CardioExcyte96 system (Nanion Technologies, Munich, Germany). The assessment was conducted under 5% O_2_ for 3 h, followed by 1% O_2_ for 7 h. All cardiomyocytes were systematically recorded using the CardioExcyte Control software (Nanion Technologies, Munich, Germany).

### Detection of mitochondrial reactive oxygen species (ROS)

The fluorescent probe MitoSOX (Invitrogen, Waltham, MA, USA) was used to detect mitochondrial ROS in frozen sections of freshly harvested mouse cardiac tissues and neonatal cardiomyocytes, following the manufacturer's instructions. Images were captured using a confocal laser-scanning microscope (Olympus VS200, Tokyo, Japan). The data were analyzed using the ImageJ software.

### Determination of ATP and lactate level

The LV tissues from mice and neonatal cardiomyocytes were lysed, homogenized, and then centrifuged at 4℃. The collected supernatant was analyzed for protein concentration using a bicinchoninic acid kit according to the manufacturer's instructions. ATP levels and lactate production in the supernatant were measured using an ATP assay kit (Beyotime, Shanghai, China) and a lactate assay kit (Nanjing Jiancheng Bioengineering Institute, Nanjing, China), respectively, following the manufacturer's instructions. Measurements were taken using a microplate reader, and the results were normalized to the protein levels.

### Mitochondrial membrane potential (MMP) assay

The MMP was assessed in neonatal cardiomyocytes using a JC-1 MMP assay kit (Beyotime, Shanghai, China). Images were captured using a confocal microscope. The mean fluorescence intensity was quantified using the ImageJ software.

### Statistical analysis

Statistical analyses were performed using GraphPad Prism 8.0 (GraphPad Software Inc., La Jolla, CA, USA). Data were expressed as the mean ± standard error of the mean. A Student's t-test was used to compare two groups, while one- or two-way ANOVA was employed for three or more groups. Bonferroni post-hoc tests were conducted for further comparisons following ANOVA, when appropriate. Statistical significance was set at P < 0.05.

## Results

### Pioglitazone prevents hypoxia-induced left ventricular dysfunction

**Figure 1:**
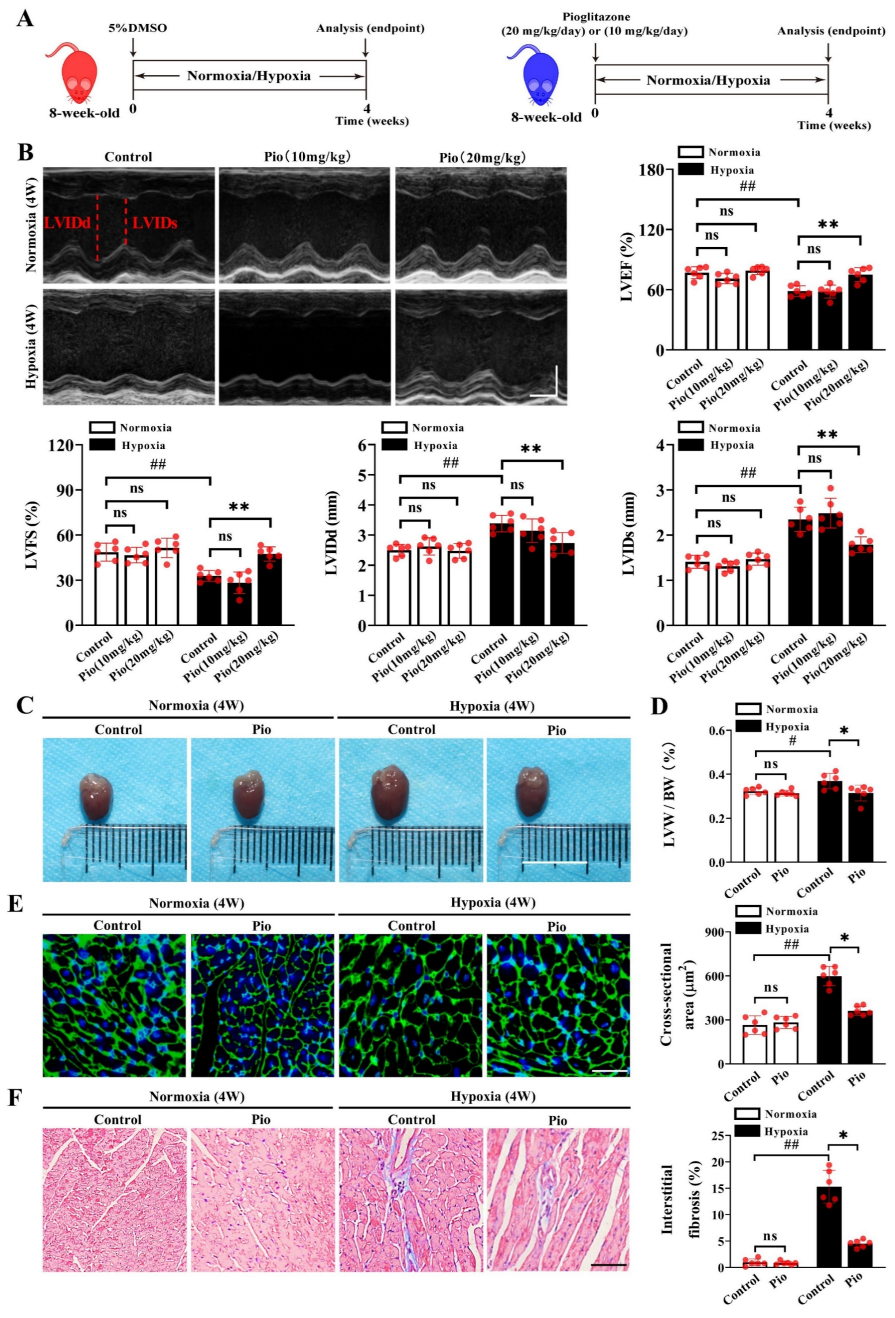
Pioglitazone prevents hypoxia-induced left ventricular dysfunction. (A) Experimental design: 8-week-old male C57BL/6J mice were randomly divided into normoxia control, normoxia pioglitazone (pio) treatment, hypoxia control, and hypoxia pioglitazone treatment groups (*n* = 6). (B) Representative echocardiographic images and data analysis of left ventricular (LV) function in control mice and mice treated with different doses of pio (10 or 20 mg/kg of body weight) (*n* = 6). LVIDd (left ventricular diastolic internal dimension) and LVIDs (left ventricular systolic internal dimension) were annotated with red dotted lines. Scale bars, vertical 2mm and horizontal 50ms. Echocardiographic data analyses the left ventricular ejection fraction (LVEF), left ventricular fractional shortening (LVFS), LVIDd and LVIDs. (C) Representative images of mice hearts in normoxic and hypoxic conditions. Scale bar, 1cm. (D) Quantification of LV hypertrophy as assessed by the ratio of LV weight to body weight (LVW/BW) (*n =* 6). (E) Representative images of wheat germ agglutinin (WGA) staining and quantification of the cross-sectional area of cardiomyocyte from the LV of the control and pio groups (*n* = 6). Scale bar, 50μm. (F) Representative images of masson trichrome staining and quantification of the interstitial fibrosis from LV of control and pio treatment groups (*n* = 6). Scale bar, 50μm. ^*^P < 0.05, ^**^P < 0.01 in control group *vs.* pio group under hypoxia. ^#^*P* < 0.05, ^##^*P* < 0.01 in control group under normoxia *vs.* hypoxia.

We established a mouse model of high-altitude hypoxia-induced cardiac dysfunction and evaluated the preventive effects of pioglitazone (Figure 1A). Echocardiographic analysis revealed no significant changes in LV function in mice, including LVEF, LVFS and LVIDs/d after 2 or 3 weeks of hypoxia treatment (Supplementary Figure 1) compared with normoxia. However, compared with normoxia treatment, the 4-week hypoxia treatment significantly impaired LV function in mice, as evidenced by decreased LVEF and LVFS, as well as increased LVIDs/d (Figure 1B and Supplementary Figure 1). Under normoxic conditions, continuous administration of pioglitazone (10 or 20 mg/kg) for 4 weeks did not affect LV function in mice (Figure 1B). Moreover, 10 mg/kg pioglitazone for 4 weeks did not alleviate hypoxia-induced LV dysfunction (Figure 1B). However, administration of 20 mg/kg of pioglitazone for 4 weeks effectively prevented the hypoxia-induced LV dysfunction, as evidenced by increased LVEF and LVFS and decreased LVIDs/d (Figure 1B).

Furthermore, LV remodeling was evaluated by measuring the LV weight/body weight ratio (LVW/BW), the cross-sectional area of cardiomyocytes, and the extent of myocardial fibrosis. Our data showed that the 4-week hypoxia treatment significantly promoted LV remodeling in control mice compared with the 4-week normoxic treatment, as evidenced by increased LVW/BW, enlarged myocardial cell cross-sectional area, and myocardial fibrosis (Figure 1C-1F). Notably, continuous administration of 20mg/kg pioglitazone for 4 weeks under hypoxic conditions resulted in significant reductions in LVW/BW, cardiomyocyte cross-sectional area, and interstitial myocardial fibrosis levels in mice, suggesting that pioglitazone may attenuate hypoxia-induced LV remodeling (Figure 1C-1F). These findings indicate that pioglitazone can prevent cardiac dysfunction induced by high-altitude hypoxia.

### Pioglitazone prevents hypoxia-induced pulmonary arterial hypertension and RV dysfunction

**Figure 2:**
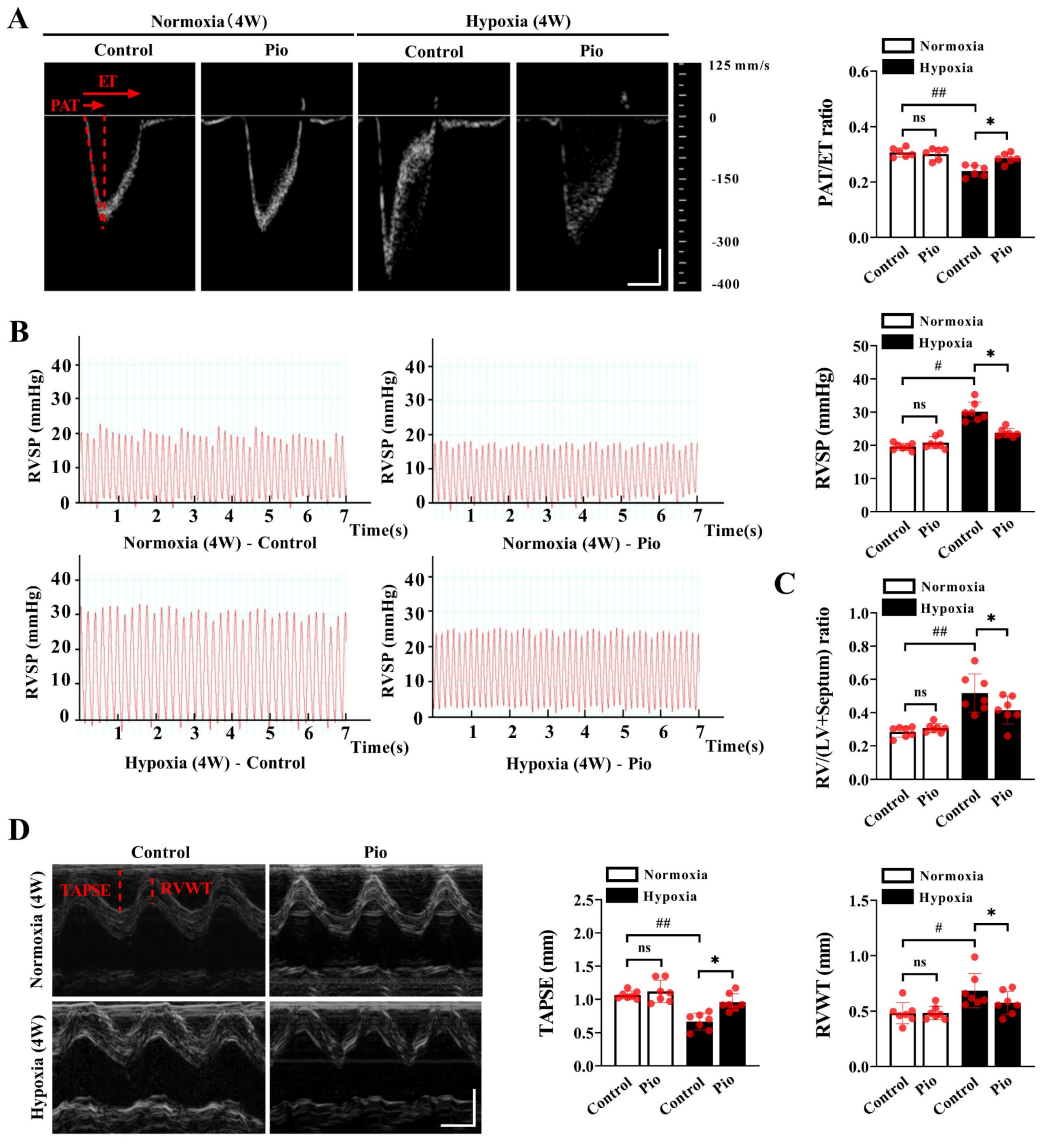
Pioglitazone prevents hypoxia-induced pulmonary arterial hypertension and right ventricular dysfunction. (A) Representative echocardiographic images of pulsed wave doppler of pulmonary artery flow and quantification of pulmonary artery acceleration time to ejection time ratio (PAT/ET ratio) in the control and pio groups under normoxic and hypoxic conditions (*n* = 6). PAT and ET were annotated with red dotted lines. Scale bars, vertical 100 mm/s and horizontal 30 ms. (B) Representative images and quantification of right ventricular systolic pressure (RVSP) in the control and pio groups (*n* = 7). (C) Quantification of right ventricular (RV) hypertrophy as assessed by the ratio of RV weight to left ventricular (LV) plus septum weight (RV/LV + Septum) (*n* = 7). (D) Representative echocardiographic images of the right ventricle (RV) from the control and pio groups (*n* = 7), accompanied by the quantification of tricuspid annular plane systolic excursion (TAPSE) and right ventricular wall thickness (RVWT). TAPSE and RVWT were annotated with red solid lines. Scale bars, vertical 2 mm and horizontal 50 ms. ^*^P < 0.05, ^**^P < 0.01 in control group *vs.* pio group under hypoxia. ^#^*P* < 0.05, ^##^*P* < 0.01 in control group under normoxia *vs.* hypoxia.

To investigate the factors that trigger pathological changes in the LV, we evaluated the development of PH and RV systolic function under high-altitude hypoxia. Our data demonstrated that mice exposed to hypoxia for 4 weeks showed a decrease in the PAT/ET ratio, an increase in RVSP and RV/LV+ Septum compared with those exposed to normoxia (Figure 2A-2C), indicating that chronic hypoxia promoted the development of PH. However, the mice treated with continuous administration of 20mg/kg pioglitazone under hypoxic conditions for 4 weeks exhibited an increase in the PAT/ET ratio, a decrease in RVSP and RV/LV+ Septum compared with the control mice (Figure 2A-2C). These findings indicate that pioglitazone attenuate hypoxia-induced PH. Additionally, our data showed that mice subjected to 4 weeks of hypoxia exhibited decreased TAPSE and increased RVWT compared with those subjected to normoxia (Figure 2D). However, under hypoxic conditions, treatment of mice with pioglitazone resulted in an increase in TAPSE and a decrease in RVWT compared with the control group (Figure 2D), indicating that pioglitazone can alleviate hypoxia-induced RV systolic dysfunction and hypertrophy. These findings suggest that pioglitazone has the potential to alleviate PH and related cardiac pathological changes induced by chronic high-altitude hypoxia.

### Pioglitazone enhances cardiomyocyte contractility and induces changes in cardiomyocyte electrophysiological activity under hypoxic conditions

**Figure 3:**
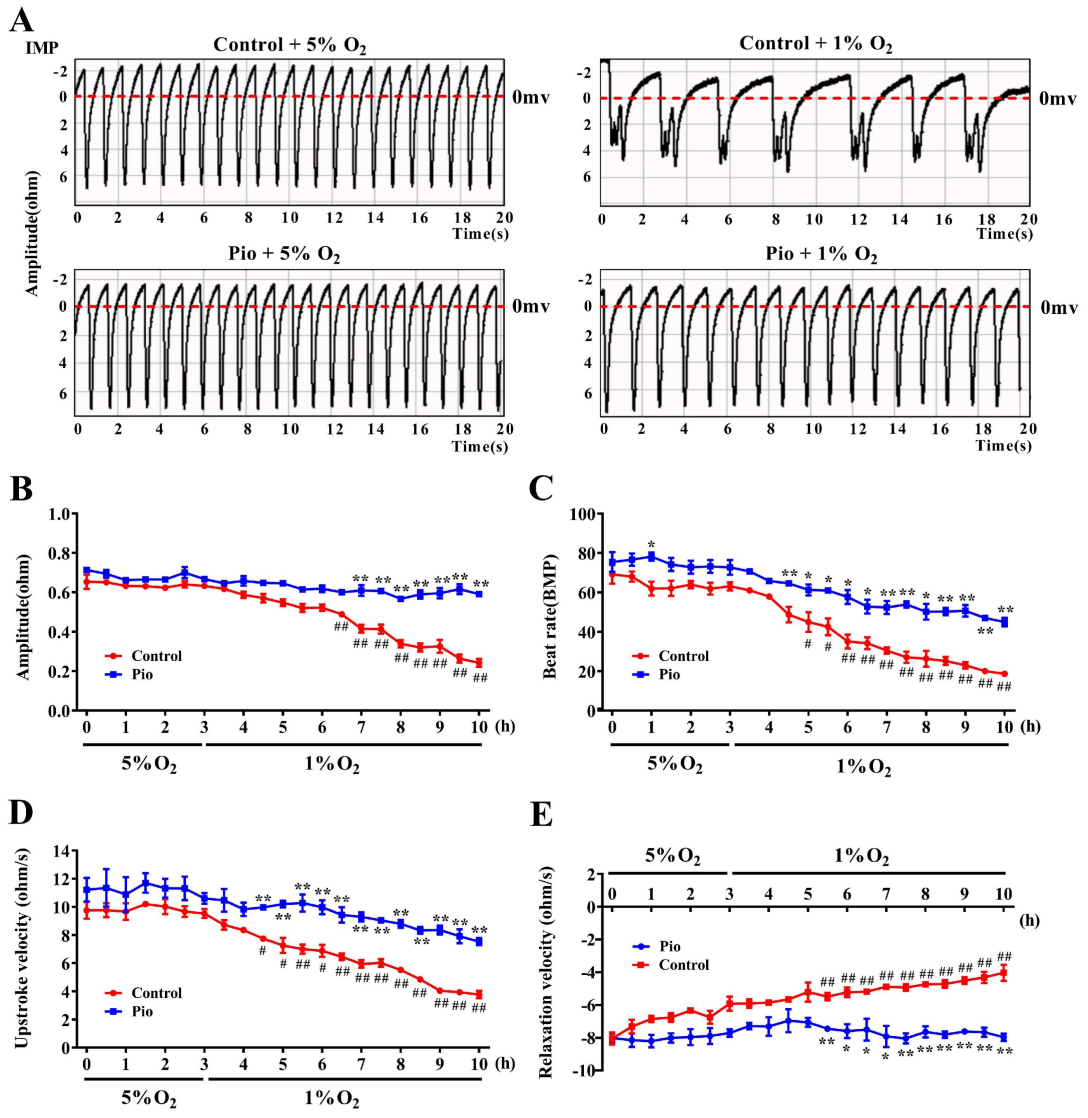
Pioglitazone promotes cardiomyocyte contractility under hypoxia. (A) Representative tracings of cardiomyocyte IMP in the control and pio groups under 5% O_2_ and 1% O_2_. (B - E) Recordings of IMP amplitude (B), IMP beat rate (C), IMP upstroke velocity (D), and IMP relaxation velocity (E) in control and pio cardiomyocytes. The recordings were obtained over a period of 3 hours under 5% O_2_ and subsequently 7 hours under 1% O_2_. Data were from three independent experiments. ^*^P < 0.05, ^**^P < 0.01 in control *vs*. pio group under 1% O_2_. ^#^*P* < 0.05, ^##^*P* < 0.01 in control group under 5% O_2_ *vs*. 1% O_2_.

To further elucidate the mechanisms by which pioglitazone prevents cardiac dysfunction under high-altitude hypoxia, we cultured neonatal rat cardiomyocytes and evaluated the effects of pioglitazone on cardiomyocyte contractility under hypoxia by measuring cardiomyocyte IMP and EFP. The median oxygen level in peripheral tissues is approximately 5%, but drops to approximately 1% during pathological hypoxia^26^,^27^. To evaluate the contractility of cardiomyocytes under physiological normoxic (5% O_2_) and pathological hypoxic conditions (1% O_2_), we measured cardiomyocyte IMP, including IMP amplitude, beat rate, upstroke velocity (systolic rate), and relaxation velocity (diastolic rate). As shown in Figure 3, under 5% O_2_, there were no significant differences in IMP amplitude, upstroke velocity, or relaxation velocity between the control and pioglitazone-treated groups of cardiomyocytes, although the pioglitazone-treated group had a slightly higher beat rate than the control group. Conversely, under 1% O_2_, the pioglitazone-treated group exhibited significantly higher IMP amplitude, beat rate, upstroke velocity, and relaxation velocity than the control group. Additionally, the cardiomyocyte contractility in the control group significantly decreased under 1% O_2_ compared with that under 5% O_2_, whereas the pioglitazone-treated group exhibited comparable cardiomyocyte contractility under 5% O_2_ and 1% O_2_. These findings suggest that pioglitazone preserves the contractile and relaxation capacities of cardiomyocytes in pathologically hypoxic environments.

Similar to the clinical electrocardiogram (ECG), EFP represents the propagation of cardiomyocyte action potentials on the electrode array. Field potential duration (FPD) is equivalent to the organ-level QT interval on the ECG. Prolonged QT intervals increase the risk of ventricular tachycardia and torsades de pointes, which can progress to ventricular fibrillation^28^,^29^. As shown in Supplementary Figures 2A and 2B, hypoxia significantly prolonged the FPD of cardiomyocytes in the control group, indicating an increased risk of prolonged QT intervals. However, in 1% O_2_, the pioglitazone-treated group showed a reduction in the FPD of cardiomyocytes compared with the control group, suggesting that pioglitazone inhibited hypoxia-induced FPD prolongation in cardiomyocytes. These findings suggest that pioglitazone can potentially prevent hypoxia-induced QT interval prolongation. Overall, pioglitazone can prevent cardiac dysfunction caused by high-altitude hypoxia by enhancing the contractility of cardiomyocytes and reducing QT interval prolongation.

### Pioglitazone prevents cardiac dysfunction under hypoxic conditions through a non-insulin-dependent mechanism

**Figure 4:**
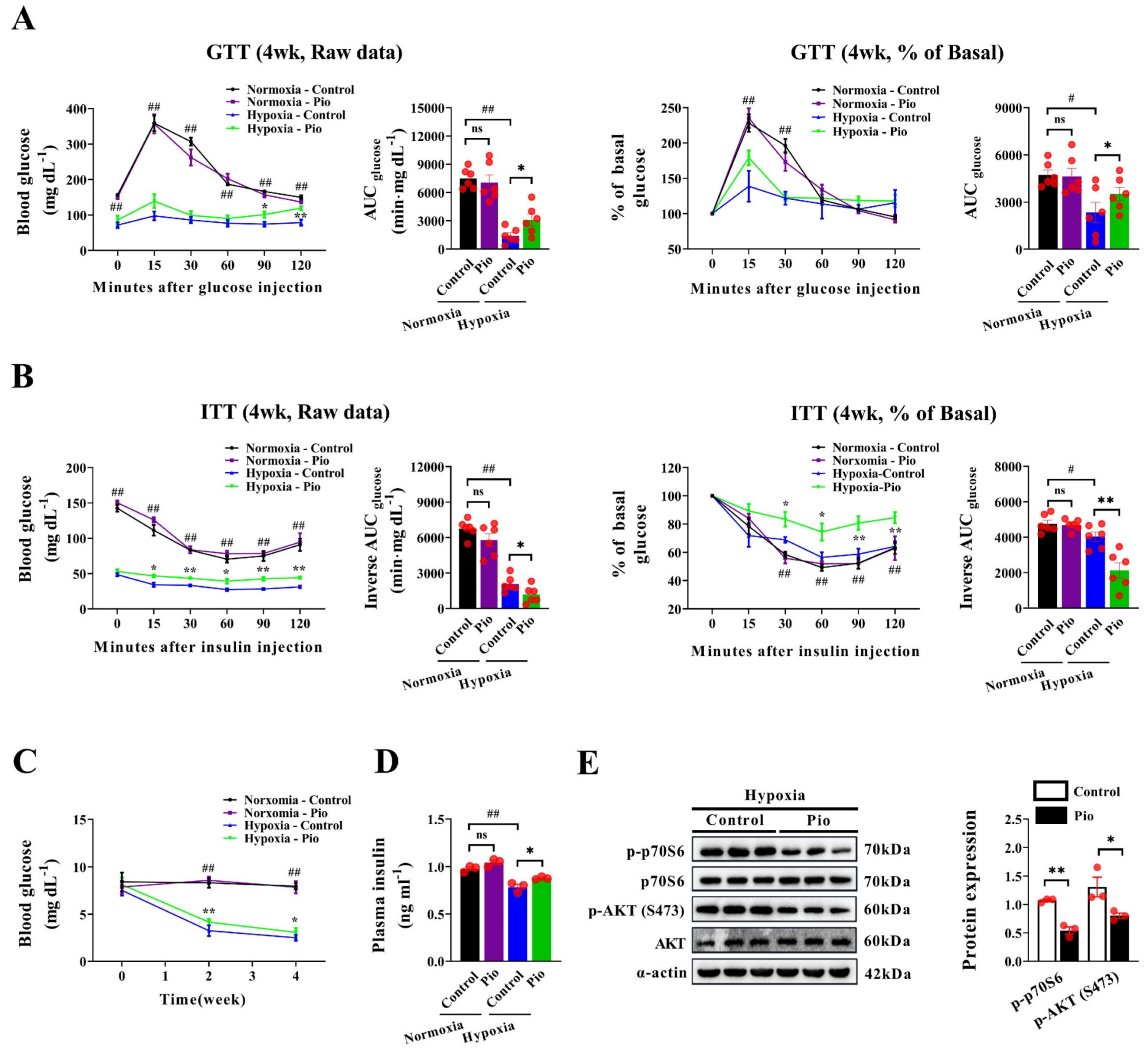
Pioglitazone prevents cardiac dysfunction under hypoxia by acting through a non-insulin signaling pathway. (A - B) The raw data of the glucose tolerance test (GTT) and insulin tolerance test (ITT), along with the percentage of basal blood glucose during GTT or ITT, were recorded for both the control and pio groups under normoxic and hypoxic conditions (*n* = 6). The corresponding area under the curve (AUC) was calculated for each mouse in both groups (*n* = 6). (C-D) Blood glucose (*n* = 6) and plasma insulin levels (*n* = 3) in control and pio groups under normoxic and hypoxic conditions. (E) Western blotting analysis of p - AKT (S473), AKT, p - p70S6 and p70S6 expression in mice heart from control and pio groups under hypoxic conditions. (*n* = 3). ^*^P < 0.05, ^**^P < 0.01 in control group *vs.* pio group under hypoxic conditions. ^#^*P* < 0.05, ^##^*P* < 0.01 in control group under normoxia *vs.* hypoxia.

Considering the insulin-sensitizing effect of 20 mg/kg pioglitazone in diabetic mice^30^, we further explored whether pioglitazone affects cardiac function in hypoxic mice by regulating insulin sensitivity. Under normoxic conditions, the GTT and ITT showed no significant differences in the AUC for GTT and the reverse AUC in the ITT between 20 mg/kg pioglitazone-treated and control mice (Figure 4A and 4B), indicating that pioglitazone did not affect plasma glucose clearance in normoxic mice. Additionally, hypoxic mice exhibited an enhanced plasma glucose clearance rate, as evidenced by the decreased AUC in the GTT, compared with normoxic mice (Figure 4A). However, in this context, the reverse AUC in the ITT was reduced owing to the lower baseline blood glucose levels observed in hypoxic mice (Figure 4B). Moreover, under hypoxic conditions, pioglitazone-treated mice exhibited a lower capacity for plasma glucose clearance than control mice, as demonstrated by an increased AUC in the GTT and a decreased reverse AUC in the ITT (Figure 4A and 4B). In addition, hypoxia decreased fasting blood glucose and plasma insulin levels in mice, however, pioglitazone partially reversed this effect (Figure 4C and 4D).

Moreover, western blotting analysis of the insulin signaling pathway revealed that under hypoxic conditions, pioglitazone-treated mice exhibited lower phosphorylated Akt and p70S6 in the myocardium than control mice (Figure 4E). These findings indicate that pioglitazone does not activate the insulin signaling pathway in the myocardium during hypoxia. Instead, pioglitazone prevents cardiac dysfunction under hypoxic conditions through a mechanism independent of insulin sensitization.

### Pioglitazone promotes ATP synthesis and inhibits ROS production under hypoxic conditions

**Figure 5:**
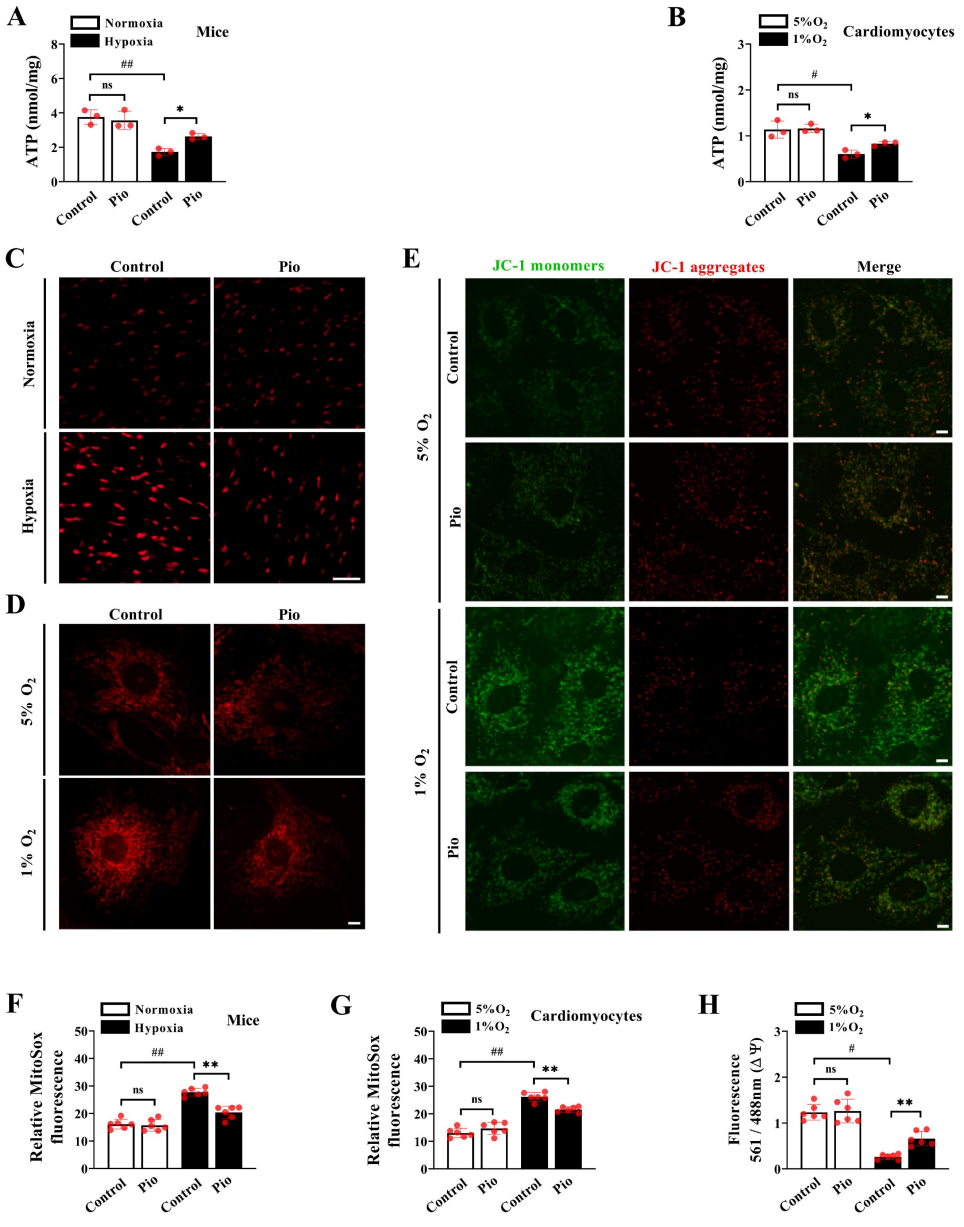
Pioglitazone promotes ATP production and inhibits ROS accumulation under hypoxia. (A) Cardiac ATP levels in mice (*n* = 3). (B) Cardiomyocyte ATP levels. Data were from three independent experiments. (C) Representative confocal microscope images of cardiac mitochondrial ROS production in control and pio mice under normoxic and hypoxic conditions (MitoSOX fluorescence; red). Original magnification×20. Scale bar, 20 μm. *n* = 6. (D) Confocal images of cells stained with MitoSOX in control and pio cardiomyocytes under 5% O_2_ and 1% O_2_. Original magnification×100. Scale bars: 5μm. *n* = 6. (E) Confocal microscope images of MMP in cardiomyocytes using JC-1 staining. The red/green fluorescent intensity ratio was analyzed. Original magnification×100. Scale bars: 5 μm. *n* = 6. (F) The quantitative analysis of cardiac mitochondrial ROS production in mice. (G) The quantitative analysis of cardiac mitochondrial ROS production in cardiomyocytes. (H) The quantitative analysis of MMP in cardiomyocytes. ^*^P < 0.05, ^**^P < 0.01 in control group *vs.* pio group under hypoxia. ^#^*P* < 0.05, ^##^*P* < 0.01 in control group under normoxia *vs*. hypoxia.

To further explore the mechanism by which pioglitazone enhances cardiomyocyte contractility under hypoxic conditions, we measured ATP levels of cardiomyocytes *in vivo* and *in vitro*. In the *in vivo* experiments, there was a significant decrease in cardiac ATP levels in control mice subjected to hypoxia for 4 weeks (Figure 5A). However, pioglitazone-treated mice showed significantly higher cardiac ATP levels than control mice under the same 4-week hypoxic conditions (Figure 5A). As expected, in the *in vitro* experiments, exposure to 1% O_2_ significantly reduced ATP levels in control cardiomyocytes, whereas pioglitazone treatment prevented hypoxia-induced inhibition of ATP levels (Figure 5B).

Considering the association between excessive ROS production and cardiac pathology^31^,^32^, we measured the mitochondrial ROS levels of cardiomyocytes *in vivo* and *in vitro*. Our data demonstrated a significant increase in cardiac mitochondrial ROS levels in control mice exposed to hypoxia for 4 weeks compared with those exposed to normoxia (Figure 5C and 5F). However, the levels of cardiac mitochondrial ROS significantly reduced in pioglitazone-treated mice compared with those in control mice during the same 4-week hypoxic period (Figure 5C and 5F). To confirm this finding, our *in vitro* data revealed that mitochondrial ROS levels were significantly higher under 1% O_2_ than those under 5% O_2_ in cardiomyocytes (Figure 5D and 5G). Nevertheless, under 1% O_2_, cardiomyocytes treated with pioglitazone exhibited decreased ROS levels compared with control cardiomyocytes (Figure 5D and 5G), suggesting that pioglitazone may effectively inhibit the excess production of cardiac mitochondrial ROS induced by hypoxia. Considering that excess ROS production was closely correlated with reduced mitochondrial function, we analyzed MMP. Treatment with 1% O_2_ significantly decreased MMP levels in control cardiomyocytes compared with treatment with 5% O_2_ (Figure 5E and 5H). However, in 1% O_2_, pioglitazone-treated cardiomyocytes exhibited significantly increased MMP levels compared with control cardiomyocytes (Figure 5E and 5H). Collectively, these results indicate that pioglitazone treatment enhances mitochondrial ATP production, reduces ROS accumulation, and improves MMP in cardiomyocytes, ultimately alleviating high-altitude hypoxia-induced cardiac dysfunction.

### Pioglitazone promotes reprogramming of cardiac glucose metabolism in hypoxic hearts

**Figure 6:**
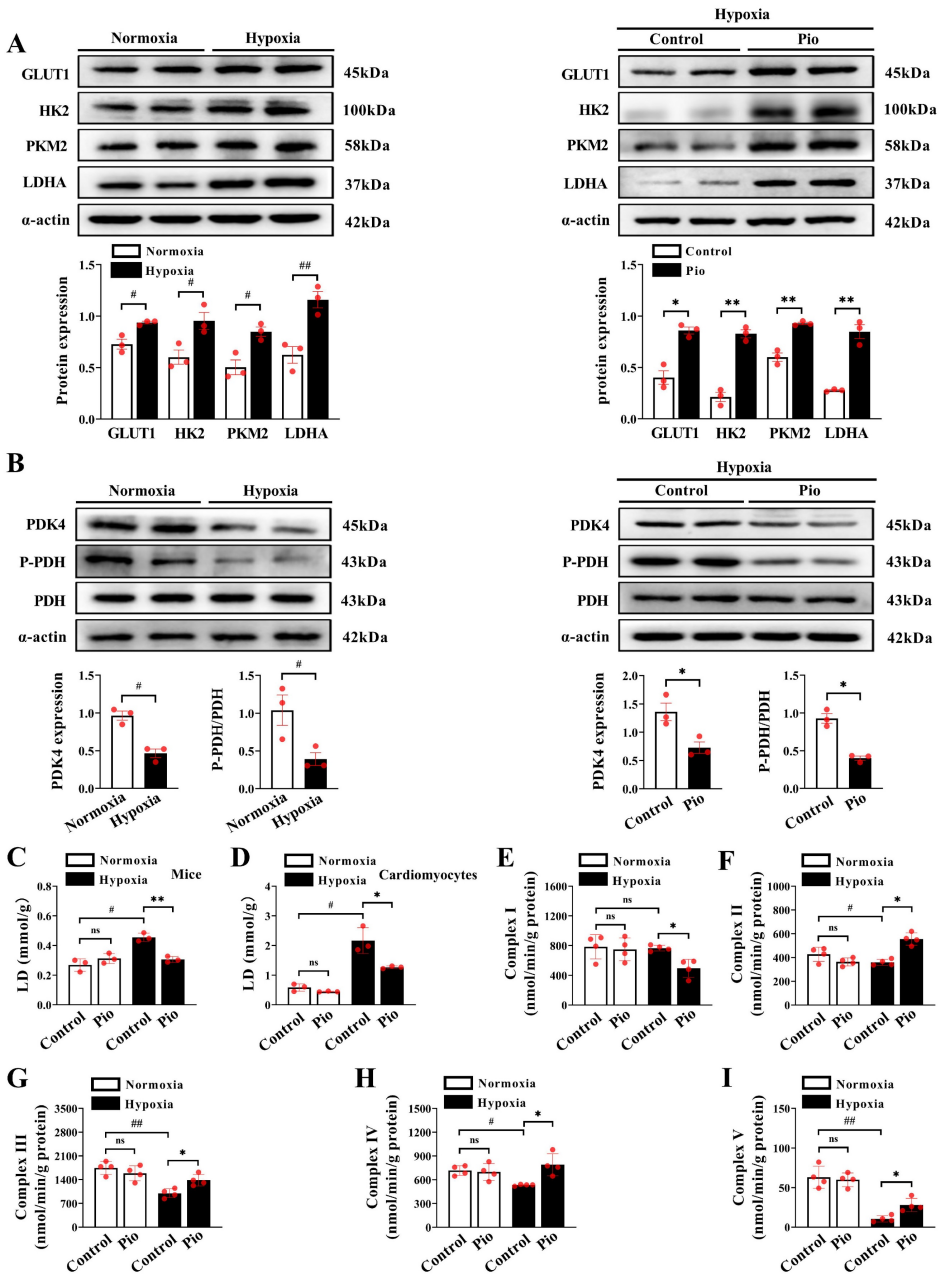
Pioglitazone enhances the capacity for glycolysis and glucose oxidation in hearts under hypoxia. (A - B) Western blotting and quantitative analyses of glycolysis and glucose oxidation - related enzymes in cardiac tissues of mice under normoxic and hypoxic conditions (*n* = 3). (C) Analysis of lactate generation in cardiac tissues of mice (*n* = 3). (D) Analysis of lactate generation in cardiomyocytes. Data were from three independent experiments. (E - I) Analysis of complexes I, II, III, IV and V activities in cardiac tissues of mice (*n* = 4). *P* < 0.05, ^**^P < 0.01 in control group *vs.* pio group under hypoxia. ^#^*P* < 0.05, ^##^*P* < 0.01 in control group under normoxia *vs.* hypoxia.

We investigated the mechanism by which pioglitazone regulates ATP production in cardiomyocytes by assessing glucose metabolism and the expression of enzymes associated with glucose metabolism using western blotting. Our results showed a significant increase in the expression of cardiac glycolysis-related enzymes, including glucose transporter protein (GLUT1), hexokinase-2 (HK2), pyruvate kinase M2 (PKM2), and lactate dehydrogenase A (LDHA) in mice exposed to hypoxia for 4 weeks compared with those exposed to normoxia (Figure 6A). Moreover, the expression levels of cardiac HK2, PKM2, and LDHA in pioglitazone-treated mice were significantly higher than those in the control group after 4 weeks (Figure 6A), suggesting that pioglitazone enhanced the glycolytic capacity of cardiomyocytes under hypoxic conditions. Pyruvate dehydrogenase (PDH) and pyruvate dehydrogenase kinase (PDK) are crucial proteins in regulating glucose oxidation. PDK functions as a kinase to phosphorylate PDH (P-PDH). This phosphorylation inhibits PDH activity, thereby decreasing glucose oxidation capacity^33^. Our data revealed a significant decrease in cardiac PDK4 expression and cardiac PDH phosphorylation level in hypoxic mice compared with those exposed to normoxia (Figure 6B). The administration of pioglitazone further suppressed the expression of PDK4 and decreased PDH phosphorylation in the hypoxic heart (Figure 6B). These findings indicate that pioglitazone enhances PDH activity in cardiomyocytes under hypoxic conditions, thereby augmenting the capacity for glucose oxidation.

Excessive lactate production increases the risk of heart failure^34^. Therefore, we further investigated the effects of pioglitazone on lactate generation *in vivo* and *in vitro*. After 4 weeks of hypoxia, control mice showed a significant increase in cardiac lactate production compared with mice under normoxic conditions (Figure 6C). However, pioglitazone administration effectively reduced hypoxia-induced cardiac lactate build-up in mice (Figure 6C). Similarly, in *in vitro* experiments, control cardiomyocytes exhibited a marked increase in lactate production when exposed to 1% O_2_ compared with 5% O_2_, however, pioglitazone administration alleviated the hypoxia-induced cardiomyocyte lactate production (Figure 6D). These findings suggest that pioglitazone has the potential to decrease lactate production during hypoxia and mitigate its harmful effects on the myocardium. Additional data revealed that the activity of mitochondrial complex I in the hearts of mice under hypoxic conditions was comparable to that observed under normoxic conditions (Figure 6E). However, treatment with pioglitazone significantly reduced the activity of complex I with that in the control group under hypoxic conditions (Figure 6E). This suggests that pioglitazone inhibits complex I activity, consequently leading to a decrease in ROS production under hypoxic conditions, which is supported by ROS primarily generated in complex I of the respiratory chain^35^. Conversely, the activities of cardiac mitochondrial complexes II, III, IV and V were significantly reduced under hypoxic conditions (Figure 6F-6I); nevertheless, treatment with pioglitazone resulted in significantly higher activities of complexes II, III, IV and V in the mice hearts than in the control group (Figure 6F-6I), indicating an enhancement of the electron transport system capacity and ATP production.

In conclusion, our findings indicate that pioglitazone improves cardiac glucose metabolism under hypoxic conditions by enhancing glycolytic capacity, promoting glucose oxidation, and optimizing electron transfer and oxidative phosphorylation processes. These adaptations result in increased ATP production and improved cardiomyocyte contractility.

### Pioglitazone prevents hypoxia-induced cardiac dysfunction by regulating the HIF-1α pathway

**Figure 7:**
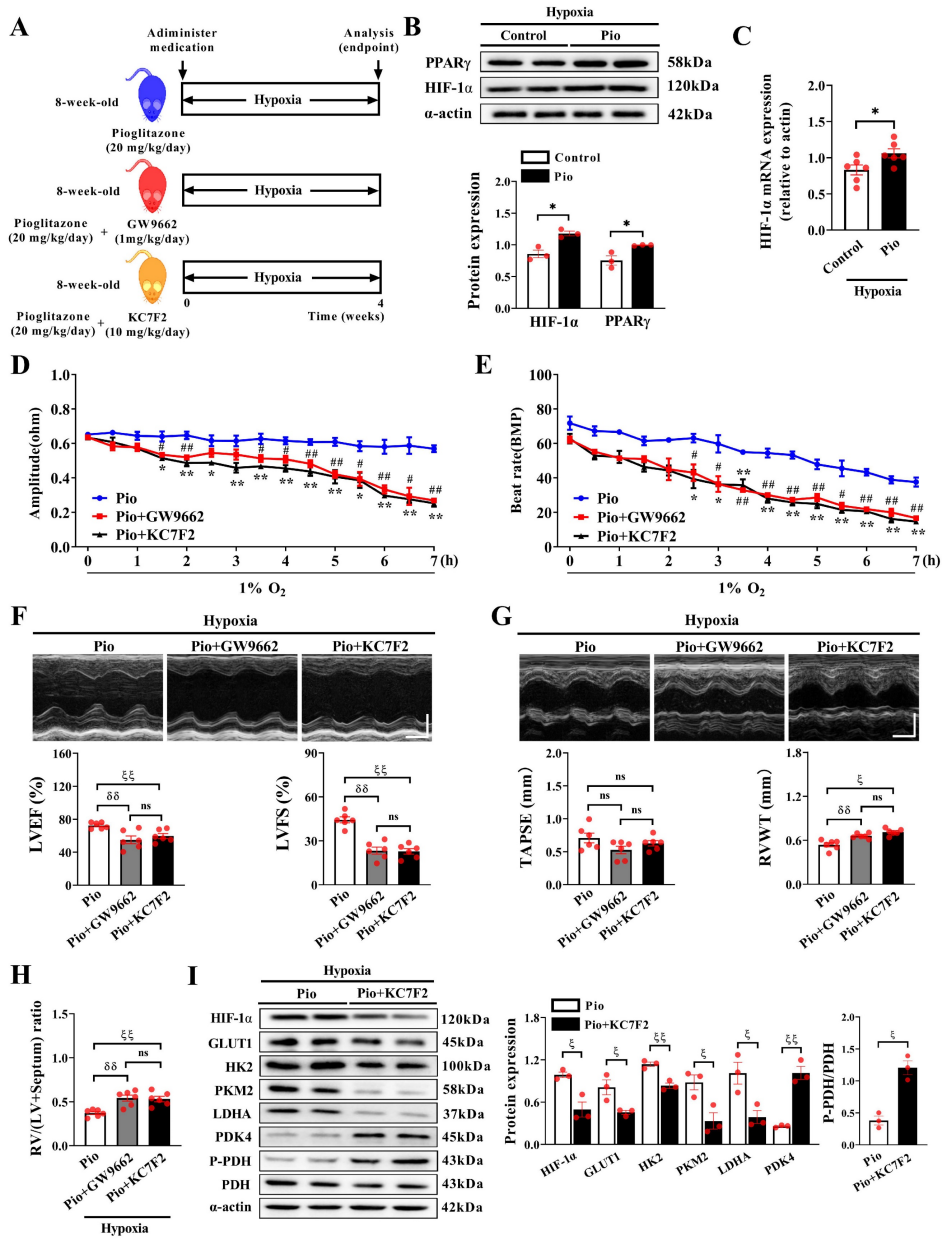
PPARγ agonist pioglitazone prevents hypoxia-induced cardiac dysfunction through modulation of the HIF-1α pathway. (A) Experimental design: 8-week-old C57BL/6J male mice were randomly divided into pio, pio combined with GW9662, and pio combined with KC7F2 groups under hypoxic conditions (*n* = 6). (B) Western blotting analysis in control and pio groups under hypoxia (*n* = 3). ^*^P < 0.05 in control group *vs.* pio group under hypoxia. (C) Quantitative real-time-polymerase chain reaction (qRT-PCR) analysis of HIF-1α mRNA expression in cardiac tissues (*n* = 6). ^*^P < 0.05 in control *vs.* pio group under hypoxia. (D-E) IMP amplitude and IMP beat rate in cardiomyocytes treated with pio, pio combined with GW9662, or pio combined with KC7F2 under 1% O_2_ for a duration of 7 hours. Data were from three independent experiments. ^*^P < 0.05, ^**^P < 0.01 in pio *vs.* pio combined with KC7F2 groups under hypoxic. ^#^*P* < 0.05, ^##^*P* < 0.01 in pio *vs.* pio combined with GW9662 groups under hypoxic. (F) Representative echocardiographic images and data analyses of LVEF and LVFS in pio, pio combined with GW9662, and pio combined with KC7F2 groups under hypoxic conditions (*n* = 6). Scale bars, vertical 2mm and horizontal 50ms. (G) Representative echocardiographic images and data analyses of TAPSE and RVWT in pio, pio combined with GW9662, and pio combined with KC7F2 groups under hypoxic conditions (*n* = 6). Scale bars, vertical 2 mm and horizontal 50 ms. (H) Quantification of RV hypertrophy as assessed by RV/LV+Septum ratio (*n* = 6). (I) Western blotting and quantitative analyses of key enzymes involved in glycolysis and glucose oxidation in cardiac tissues of mice under hypoxia (*n* = 3). (F-I; ^δδ^*P* < 0.01 in pio group *vs.* pio combined with GW9662 group under hypoxia, ^ζ^*P* < 0.05, ^ζζ^*P* < 0.01 in pio group *vs.* pio combined with KC7F2 group under hypoxia).

We further investigated whether pioglitazone reprograms cardiomyocyte glucose metabolism under hypoxic conditions by modulating the HIF-1α pathway (Figure 7A). Our data demonstrated that pioglitazone treatment significantly increased the protein and mRNA expression of HIF-1α in the hearts of mice exposed to hypoxia for 4 weeks (Figure 7B-C). Additionally, as expected, treatment with pioglitazone, a PPARγ agonist^36^, significantly increased cardiac PPARγ expression (Figure 7B). Under 1% O_2_ conditions *in vitro*, combining pioglitazone with either a PPARγ inhibitor (GW9662) or a HIF-1α inhibitor (KC7F2) significantly reduced IMP amplitude, beat rate, upstroke velocity, and relaxation velocity of cardiomyocytes, while also prolonging FPD compared with treatment with pioglitazone alone (Figure 7D and 7E, Supplementary Figure 3A-3E). These results indicate that HIF-1α, a downstream target of the PPARγ agonist pioglitazone, plays a crucial role in preventing hypoxia-induced cardiac contractile dysfunction and QT interval prolongation in cardiomyocytes.

Furthermore, under hypoxic conditions, mice treated with pioglitazone combined with GW9662 or KC7F2 exhibited a significant decrease in LVEF and LVFS compared with those treated with pioglitazone alone (Figure 7F), indicating that inhibiting HIF-1α attenuated the preventive effect of the PPARγ agonist pioglitazone on LV dysfunction. Additionally, treatment with GW9662 or KC7F2 exacerbated RV hypertrophy in pioglitazone-treated mice under hypoxic conditions, evidenced by increased RVWT and RV/LV+Septum, although it minimally impacted TAPSE (Figure 7G and 7H). However, GW9662 or KC7F2 treatment did not affect PAT/ET and RVSP in pioglitazone-treated mice (Supplementary Figure 4A and 4B), indicating that pioglitazone prevented hypoxia-induced PH independent of the PPARγ-HIF-1α pathway.

Pioglitazone exerts a specific preventive effect on hypoxia-induced cardiac dysfunction by regulating the HIF-1α pathway. Furthermore, western blotting analysis revealed that treatment with KC7F2 decreased HIF-1α expression and reduced the expression of glycolysis-related enzymes (GLUT1, HK2, PKM2, and LDHA) while increasing the expression of glucose oxidation-related enzymes (PDK4 and P-PDH) in the hearts of hypoxic mice treated with pioglitazone (Figure 7I). These findings indicate that the inhibiting HIF-1α impairs pioglitazone-induced glucose metabolism reprogramming in the hypoxic heart. In essence, the functional role of the PPARγ agonist pioglitazone in glucose metabolic reprogramming and the cardiac response to hypoxia is partially mediated by the regulation of the HIF-1α pathway.

## Discussion

Our study yielded several important results. First, pioglitazone preserves cardiac systolic and diastolic functions and inhibits hypoxia-induced cardiomyocyte hypertrophy and cardiac fibrosis through a non-insulin-dependent mechanism, effectively preventing chronic hypoxia-induced cardiac dysfunction. Second, pioglitazone promotes cardiomyocyte contractility under hypoxic conditions. Third, pioglitazone treatment reprograms glucose metabolism in hypoxic cardiomyocytes, characterized by increasing aerobic glucose oxidation and enhancing glycolytic capacity, electron transfer and oxidative phosphorylation processes while also decreasing ROS production and effectively maintaining MMP and ATP generation in cardiomyocytes. Additionally, our data demonstrates that pioglitazone's functional role in the glucose metabolic reprogramming and cardiac response to hypoxia, at least in part, is mediated by regulating the HIF-1α pathway.

Acute exposure to high altitudes results in decreased arterial oxygen saturation, increased minute ventilation, and elevated heart rate and cardiac output, partially compensating for the reduced oxygen availability^37^-^39^. With prolonged exposure, cardiac output returns to baseline levels, but the heart rate remains elevated to compensate for the decreased stroke volume^40^. However, a prolonged elevated heart rate can contribute to cardiac overload, arrhythmias, and increased oxygen demand, potentially exacerbating myocardial hypoxia. Furthermore, chronic hypoxia can lead to pulmonary vasoconstriction and the remodeling of pulmonary arterioles, thereby increasing pulmonary vascular resistance and the development of PH^41^,^42^. PH increases ventricular overload, which requires increased myocardial oxygen demand for normal cardiac function. However, cardiopulmonary abnormalities and low oxygen levels further contribute to myocardial hypoxia and impaired cardiac function. Studies have reported that healthy individuals experienced decreased cardiac mass, diastolic function, and phosphocreatine/adenosine triphosphate (PCr/ATP) ratio within a few days of returning from the Everest Base Camp^43^. Similarly, our study indicated that chronic high-altitude hypoxia induced the development of PH, impaired cardiac contractile function, and promoted cardiac remodeling in mice. Cellular experiments demonstrated that hypoxia reduced the contractility of neonatal primary cardiomyocytes, indicating that chronic high-altitude hypoxia can result in cardiac dysfunction. However, extensive research on drug strategies for preventing high-altitude cardiac dysfunction is lacking.

Sildenafil and acetazolamide are commonly used for high-altitude illnesses. Sildenafil inhibits phosphodiesterase-5, promotes vasodilation and alleviates high-altitude pulmonary hypertension^10^. However, it does not improve cardiac function or exercise capacity in patients with HFpEF^11^. Acetazolamide inhibits carbonic anhydrase and promotes bicarbonate excretion and metabolic acidosis. This compensates for the respiratory alkalosis caused by excessive ventilation at high altitudes, preventing acute mountain sickness^8^,^9^. However, the potential side effects of acetazolamide include increased urine production and changes in taste perception^8^,^9^. Its effectiveness in preventing high-altitude cardiac disease remains unclear. Therefore, the options for safe and effective drugs to prevent high-altitude cardiac diseases are limited. Pioglitazone, an insulin sensitizer, has shown significant benefits in improving plasma glucose levels and controlling blood pressure and lipid management^15^. It enhances insulin sensitivity in obese and diabetic animal models, as well as in patients with T2DM^15^. Additionally, pioglitazone can reduce the risk of myocardial infarction and stroke and improve LV diastolic and systolic function in individuals with T2DM^16^,^17^. Pioglitazone has no negative effects on a healthy myocardium^19^. However, in severe myocardial dysfunction or advanced HF, pioglitazone may cause fluid retention and worsen HF symptoms^44^, indicating that pioglitazone administration in the pre-HF stage may potentially decrease the risk of HF development. Similarly, we found that pioglitazone prevented high-altitude hypoxia-induced myocardial dysfunction in mice. Cellular experiments demonstrated that pioglitazone enhanced the contractility of neonatal primary cardiomyocytes in hypoxic mice. Our study showed that pioglitazone is an important intervention strategy for preventing high-altitude cardiac diseases. However, further validation in high-altitude hypoxic populations is required to confirm these conclusions.

The requirement for energy to fuel optimal function increases as the heart contracts continuously. High-energy phosphate storage within cardiomyocytes is limited; thus, tight coupling of ATP production and myocardial contraction is essential for normal cardiac function^45^. Under normoxic conditions, the heart primarily relies on the oxidation of fatty acids to produce ATP, accounting for 60-90% of its energy production. However, under hypoxic conditions, the heart undergoes metabolic adaptation and relies more on glucose utilization and less on fatty acid oxidation^46^,^47^. This is due to the more efficient nature of glucose oxidation, which generates 53.7% more high-energy phosphate bonds per mole of glucose than fatty acids. Consequently, the heart produces more energy with the same amount of oxygen, thereby maintaining its function^46^,^48^. Sherpas, known for their remarkable physical performance at high altitudes, optimize their body energetics by increasing their utilization of high-efficiency oxidative fuel substrates, such as carbohydrates^13^,^14^. In response to hypoxia, the fetal heart heavily relies on glycolysis (44%) as the primary pathway for ATP production, while fatty acid oxidation contributes minimally (13%) to the overall synthesis of myocardial ATP^49^. This adaptation is consistent with the Warburg effect, where in organisms switch from oxidative metabolism to glycolysis under hypoxic conditions to maintain adequate ATP levels^50^. These studies suggest that reprogramming of glucose metabolism may play a crucial regulatory role in the myocardial response to hypoxia. Our study revealed that pioglitazone enhanced the expression of critical glycolytic enzymes, including GLUT1, HK2, and PKM2, in the myocardium of mice subjected to high-altitude hypoxia and reduced PDH phosphorylation levels. This significantly increased myocardial glycolysis and glucose oxidation, ultimately enhancing metabolic efficiency and increasing ATP levels in the hypoxic myocardium. Additionally, treatment with pioglitazone did not increase insulin sensitivity in mice under hypoxic conditions, indicating that the preventive effect of pioglitazone against hypoxia-induced cardiac dysfunction may be mediated by mechanisms other than its insulin-sensitizing properties. HIF-1α emerges as a pivotal regulator in metabolic dynamics under hypoxic circumstances^51^,^52^. Our study further elucidated that the PPARγ agonist pioglitazone could boost HIF-1α expression in the heart during hypoxia. The HIF-1α inhibitor, KC7F2, noticeably diminished glycolysis and glucose oxidation, effectively stifling the advantageous effects of pioglitazone on cardiac function and the metabolic adaptation of cardiomyocytes under hypoxic conditions. These findings indicate that pioglitazone, as a PPARγ agonist, influences metabolism and function of cardiomyocyte by modulating the HIF-1α pathway. However, the intricate regulatory interactions between PPARγ and HIF-1α in cardiomyocytes under hypoxic conditions require further investigation.

ROS plays an important pathological role in cardiovascular diseases. For example, ROS overload contributes to the opening of permeability transition pores, leading to mitochondrial and cardiomyocytes damage^32^,^53^. Consistently, our study demonstrated that hypoxia induced excessive ROS production in cardiomyocytes, decreasing MMP. Nonetheless, pioglitazone treatment effectively inhibited the excessive production of ROS in cardiomyocytes under hypoxic conditions, thereby preventing a decrease in MMP. Mitochondria, the largest consumers of myocardial oxygen, are greatly affected by the limited availability of oxygen. ROS is intricately associated with mitochondrial function, as oxidative phosphorylation inherently generates ROS as superoxide anions (O_2_^•-^) as byproducts^54^. The main site of ROS production is the respiratory chain complex I^55^. Our study revealed that, under hypoxic conditions, pioglitazone treatment effectively suppressed complex I activity, leading to a reduction in ROS production. Furthermore, pioglitazone enhanced the activity of complexes II, III, IV and V, promoting ATP production and thus contributing to the maintenance of cardiomyocyte contractility under hypoxic conditions. Therefore, pioglitazone regulates the activity of respiratory chain complexes, optimizing electron transfer and oxidative phosphorylation, thereby maintaining the function of mitochondria and cardiomyocytes under hypoxic conditions. However, the mechanisms underlying the regulation of the respiratory chain complexes by pioglitazone remain unclear. Further studies are required to enhance our understanding in this field. Despite this limitation, we believe that our study provides important new insights into the potential role of pioglitazone in the prevention of myocardial diseases at high altitudes.

In conclusion, our study provides supportive evidence that pioglitazone, through its non-insulin-sensitizing effects, promotes glucose metabolic reprogramming in cardiomyocytes, thereby enhancing their contractility and relaxation abilities and preventing high-altitude hypoxia-induced cardiac dysfunction. These findings suggest that pioglitazone may be an important pharmacological option for preventing myocardial diseases at high altitudes.

## Supplementary Material

Supplementary figures and tables.

## Abbreviations

PPARγ: peroxisome proliferator-activated receptor γ
HIF-1α: hypoxia-inducible factor 1α
PH: pulmonary hypertension
HF: heart failure
PDE-5: phosphodiesterase type-5
HFpEF: heart failure with preserved ejection fraction
T2DM: type 2 diabetes mellitus
LV: left ventricular
DMSO: dimethyl sulfoxide
LVEF: LV ejection fraction
LVFS: LV fractional shortening
LVIDs/d: LV systolic and diastolic internal dimension
RV: right ventricular
RVWT: RV wall thickness
RVID: RV internal diameter
TAPSE: tricuspid annular plane systolic excursion
PAT: pulmonary artery acceleration time
ET: ejection time
RVSP: right ventricular systolic pressure
RV/LV+Septum: weight ratio of RV to left ventricle plus septum
WGA: wheat germ agglutinin
qRT: quantitative real-time
PCR: polymerase chain reaction
GTTs: glucose tolerance tests
ITTs: insulin tolerance tests
AUC: area under the curve
IMP: impedance
EFP: extracellular field potential
MMP: mitochondrial membrane potential
LVW/BW: left ventricular weight/body weight ratio
ECG: electrocardiogram
FPD: field potential duration
GLUT1: glucose transporter protein
HK2: hexokinase-2
PKM2: pyruvate kinase M2
LDHA: lactate dehydrogenase A
PDH: pyruvate dehydrogenase
P-PDH: phosphorylate PDH
PDK: pyruvate dehydrogenase kinase
PCr/ATP: phosphocreatine/adenosine triphosphate
O_2_^•-^: superoxide anions
