# Supplementary material for: PPARγ Agonist Pioglitazone Prevents Hypoxia-induced Cardiac Dysfunction by Reprogramming Glucose Metabolism
Source: Int J Biol Sci. 2024 Aug 6;20(11):4297–313. doi: 10.7150/ijbs.98387 (PMC11379067; doi:10.7150/ijbs.98387)
Supplement: Supplementary file 1 — Supplementary figures and tables. [file ijbsv20p4297s1.pdf]

Supplemental Materials

PPARγ Agonist Pioglitazone Prevents Hypoxia-induced Cardiac Dysfunction By Reprogramming Glucose Metabolism

Supplementary

Tables 1-2 and Figures 1-4

Yijin Wang[1†], Ru Zhang[2†], Qian Chen[1], Zhangwen Lei[3], Caiyu Shi[1], Yifei Pang[3], Shan'an Zhang[4], Linjie He[5], Longtao Xu[4], Jinliang Xing[5*], Haitao Guo[5*]

[1]College of Life Sciences, Northwest University, Xi'an, 710069, China

[2]The Key Laboratory of Aerospace Medicine, Ministry of Education, Fourth Military Medical University, Xi'an, 710069, China

[3]School of Medicine, Northwest University, Xi'an, 710069, China

[4]College of Basic Medicine, Fourth Military Medical University, Xi'an, 710032, China

[5]State Key Laboratory of Cancer Biology and Department of Physiology and Pathophysiology, Fourth Military Medical University, Xi'an, 710032, China

[†]This represents these authors contributed equally to this work.

*Correspondence to: Haitao Guo and Jinliang Xing, State Key Laboratory of Cancer Biology and Department of Physiology and Pathophysiology, Fourth Military Medical University, 169 Changle West Road, Xi'an 710032, China. Email: haitaoguo@fmmu.edu.cn and xingjl@fmmu.edu.cn.

Keywords: Hypoxia, Cardiac dysfunction, Pioglitazone, Glucose metabolic reprogramming, PPARγ, HIF-1α

Supplementary Tables

Supplementary Table 1

Primary antibodies used for western blotting

**Antibody**	**Target species**	**Working dilutions**	**Catalog No.**	**Supplier**
GLUT1	Rabbit	WB: 1/1000	A6982	ABclonal, Wuhan, China
HK2	Rabbit	WB: 1/1000	A20829	ABclonal, Wuhan, China
PKM2	Rabbit	WB: 1/1000	A0268	ABclonal, Wuhan, China
LDHA	Rabbit	WB: 1/1000	A1146	ABclonal, Wuhan, China
PDH	Rabbit	WB: 1/1000	ab168379	Abcam, Cambridge, UK
P-PDH	Rabbit	WB: 1/1000	ab177461	Abcam, Cambridge, UK
PDK4	Rabbit	WB: 1/1000	A13337	ABclonal, Wuhan, China
HIF-1α	Rabbit	WB: 1/1000	ab179483	Abcam, Cambridge, UK
PPARγ	Rabbit	WB: 1/1000	16643-1-AP	Proteintech, Wuhan, China
α-actin	Rabbit	WB: 1/3000	A2319	ABclonal, Wuhan, China
AKT	Rabbit	WB: 1/1000	AP0140	ABclonal, Wuhan, China
p-AKT	Rabbit	WB:1/1000	4060	CST, Boston, USA
p-70S6	Rabbit	WB:1/1000	2708	CST, Boston, USA
p-p70S6	Mouse	WB:1/1000	9206	CST, Boston, USA

Supplementary Table 2

Primer sequences used in RT-PCR

**Gene**		**Sequences**
HIF-1α (mouse)	Forward	GCGAGAACGAGAAGAAAAGATG
	Reverse	GTGGCAACTGATGAGCAAGC
actin (mouse)	Forward	AACAGTCCGCCTAGAAGCAC
	Reverse	CGTTGACATCCGTAAAGACC

Supplementary Figures

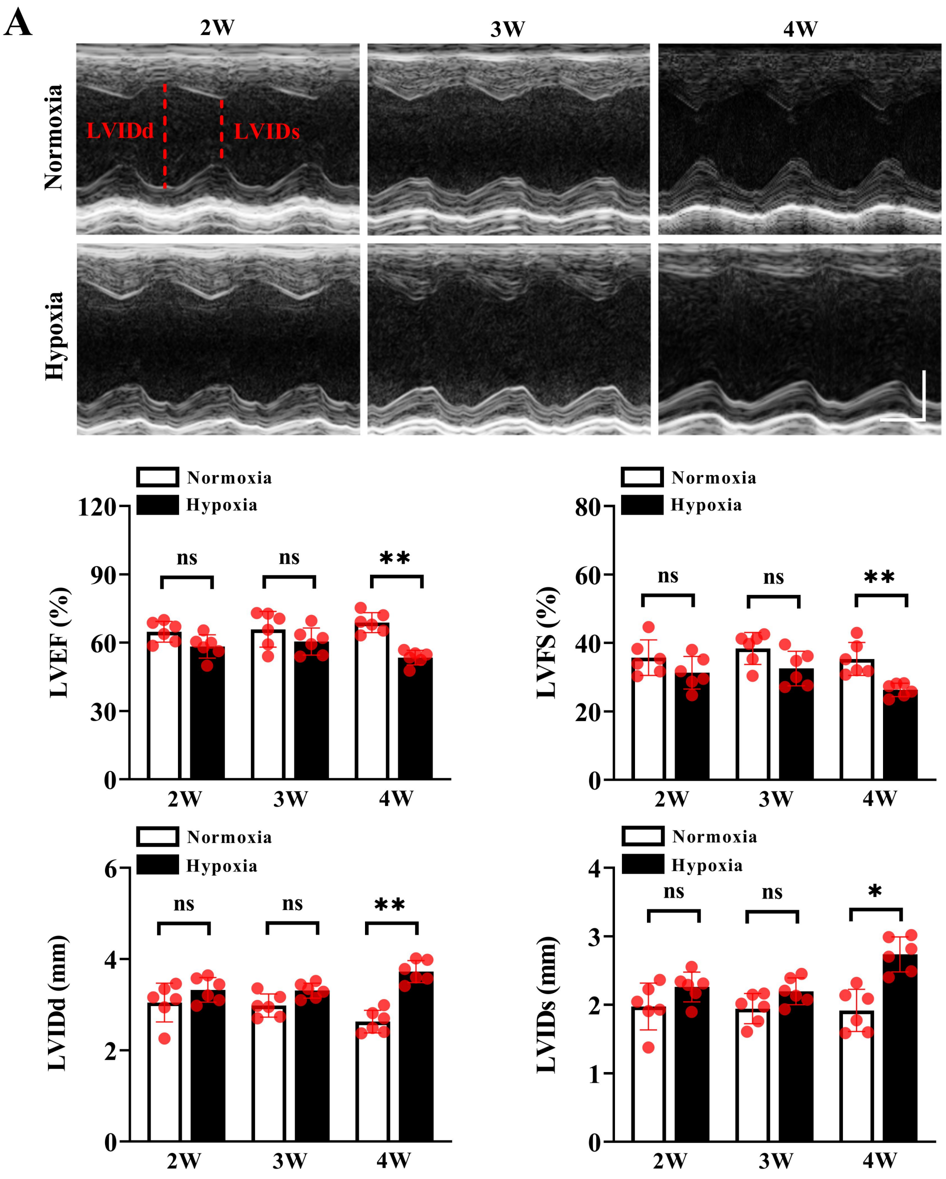

Supplementary Figure 1. Pioglitazone prevents hypoxia-induced cardiac left ventricular dysfunction. (A) Representative echocardiographic images and data analysis of left ventricular (LV) ejection fraction (LVEF), LV fractional shortening (LVFS), and LV systolic and diastolic internal dimension (LVIDs/d) under normoxic and hypoxic conditions (*n* = 6). LVIDd and LVIDs were annotated with red dotted lines. Scale bars, vertical 2mm and horizontal 50ms. $^{*}P < 0.05$, $^{**}P < 0.01$ in control group under normoxia *vs*. hypoxia.

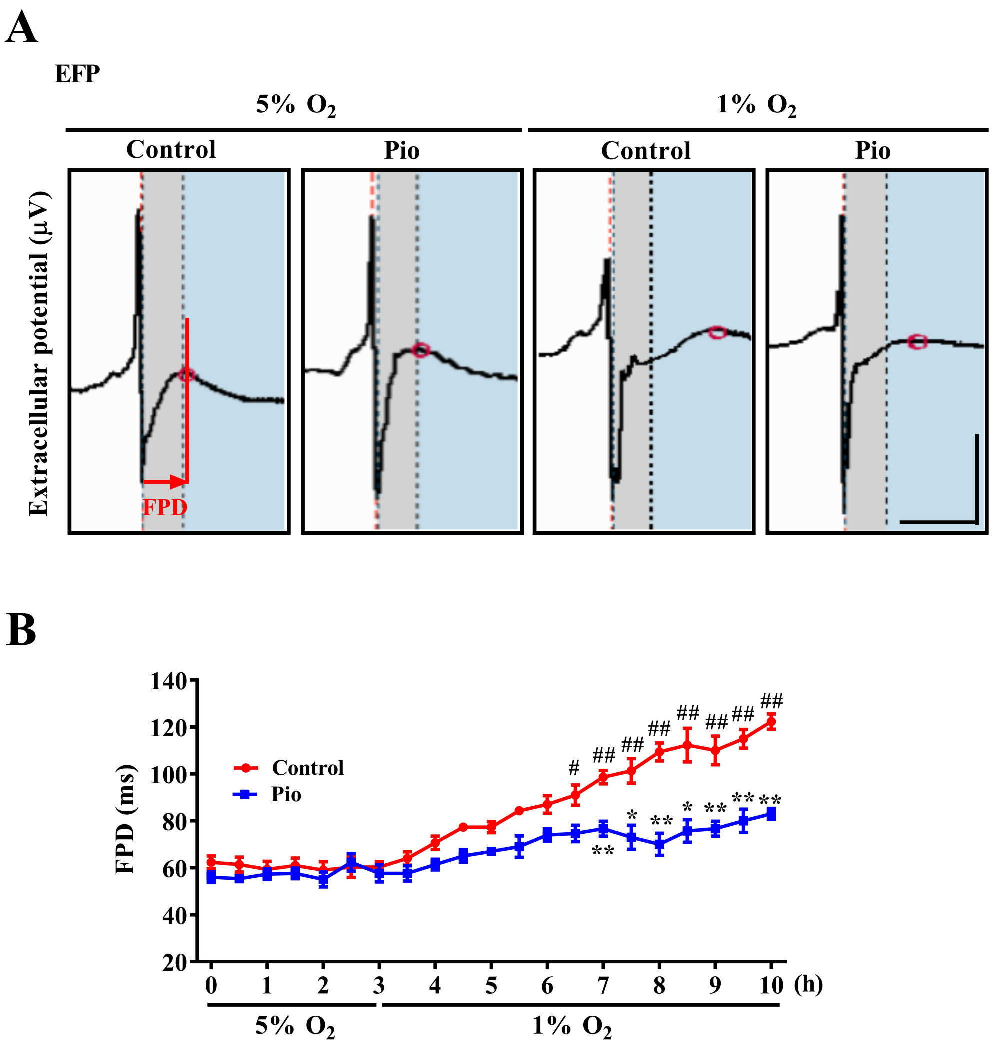

Supplementary Figure 2. Effect of pioglitazone on the extracellular field potential signal in cardiomyocytes under hypoxia. (A) Representative field potential wave tracings in control and pioglitazone (pio)-treated cardiomyocytes under 5% O_2 and 1% O_2. The field potential duration (FPD) was labeled with red arrows. Scale bars, vertical 20μV and horizontal 150ms. (B) The recordings of FPD in cardiomyocytes. Data were from three independent experiments. $^{*}P < 0.05$, $^{**}P < 0.01$ in control group *vs*. pio group under 1% O_2. $^{\#}P < 0.05$, $^{\#\#}P < 0.01$ in control group under 5% O_2 *vs*. 1% O_2.

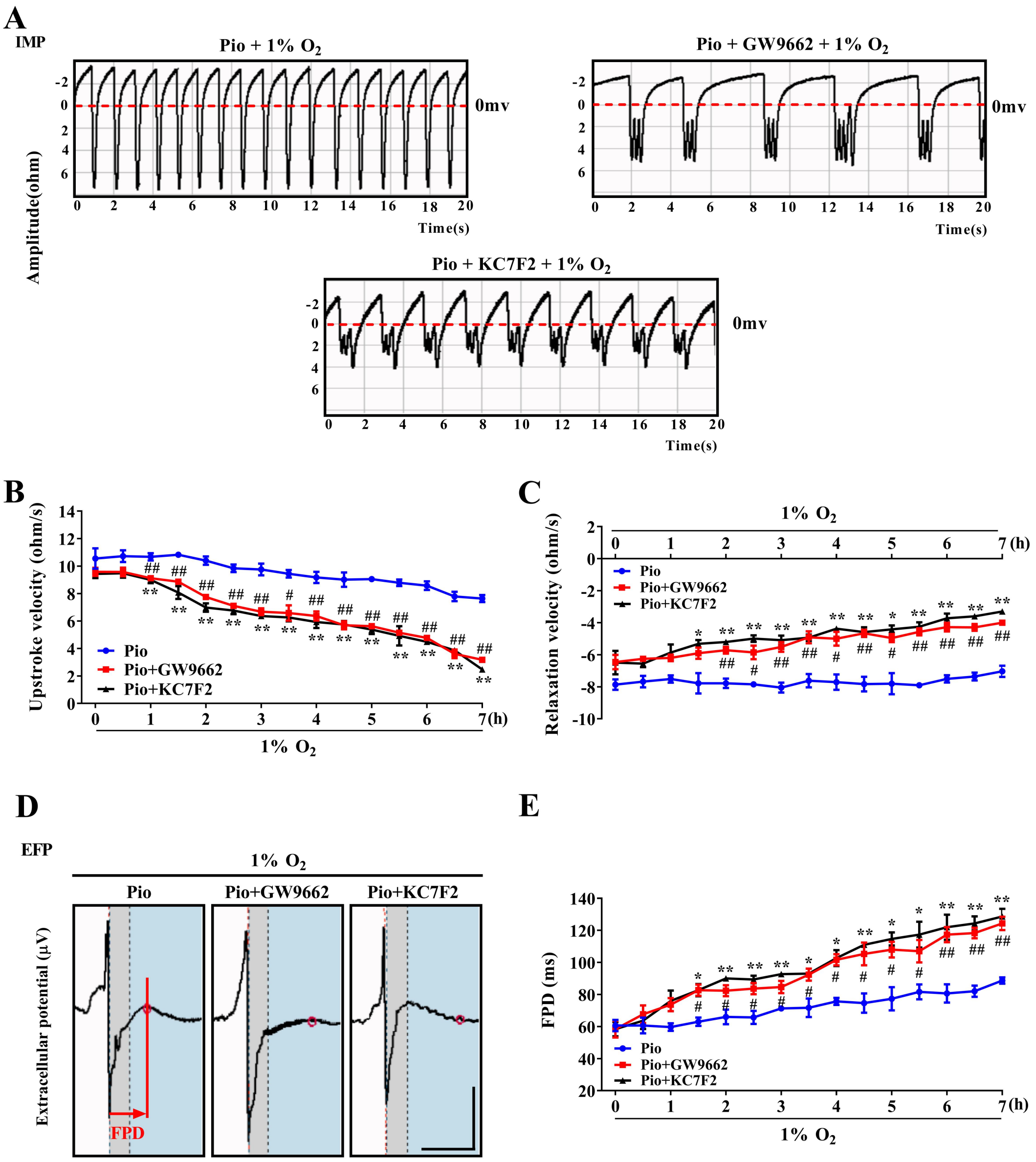

Supplementary Figure 3. The PPARγ agonist pioglitazone promotes cardiomyocyte contractility and induces changes in cardiomyocyte electrophysiological activity under hypoxia by modulating the HIF-1α pathway. (A) Representative tracings of intracellular membrane potential (IMP) in cardiomyocytes treated with pio alone, pio combined with GW9662, and pio combined with KC7F2 under 1% O_2. (B-C) Recordings of IMP upstroke velocity (B) and IMP relaxation velocity (C) in cardiomyocytes with treatment as indicated. The recordings were obtained for 7 hours under 1% O_2. (D) Representative field potential

wave tracings in cardiomyocytes with treatment as indicated. Field potential duration (FPD) was labeled. Scale bars, vertical 20μV and horizontal 150ms. (E) Recordings of FPD. Data were from three independent experiments. $^{*}P < 0.05$, $^{**}P < 0.01$ in pio group *vs.* pio combined with KC7F2 group under hypoxia. $^{\#}P < 0.05$, $^{\#\#}P < 0.01$ in pio group *vs.* pio combined with GW9662 group under hypoxia.

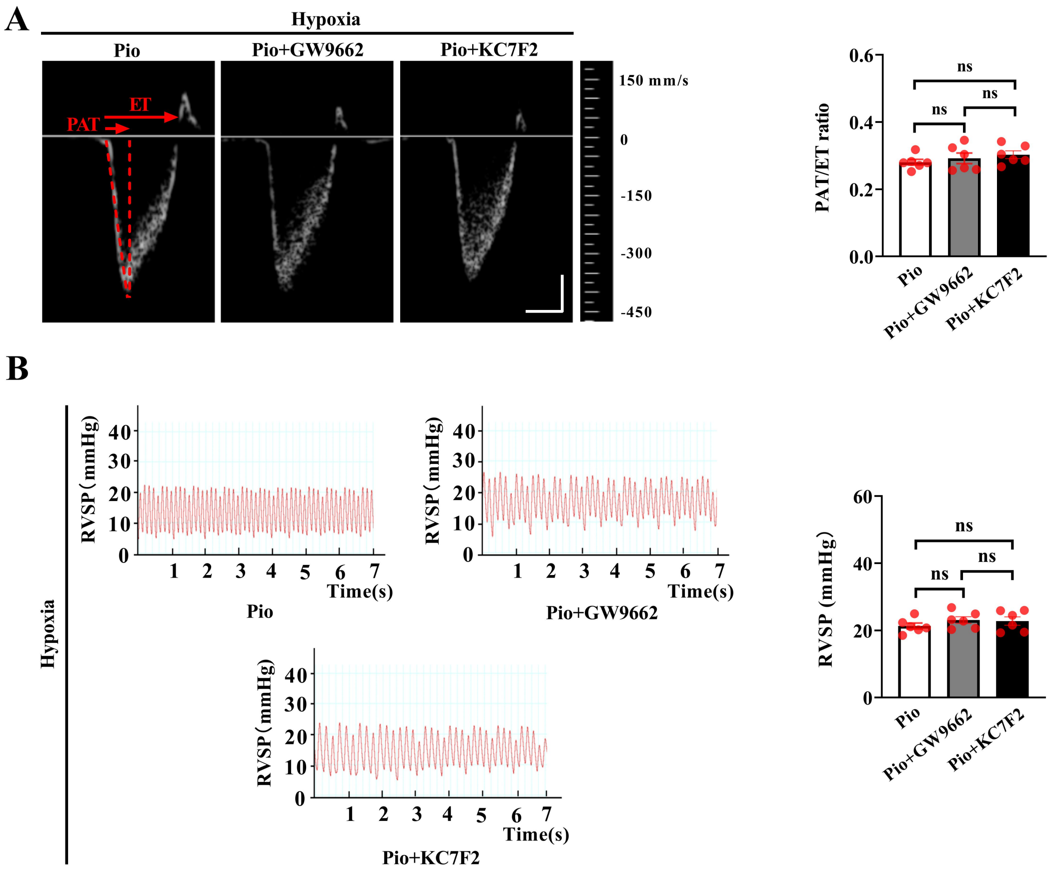

Supplementary Figure 4. Pioglitazone prevents hypoxia-induced pulmonary hypertension (PH) independently of the PPARγ-HIF-1α pathway. (A) Representative echocardiographic images of pulsed-wave doppler of pulmonary artery flow in mice treated with pio and pio combined with GW9662 or KC7F2 under hypoxia, as well as the quantification of the pulmonary artery acceleration time/ejection time ratio (PAT/ET ratio) ($n = 6$). PAT and ET were annotated with red dotted lines. Scale bars, vertical 100 mm/s and horizontal 30 ms. (B) Representative images and the quantification of right ventricular systolic pressure (RVSP) in mice with treatment as indicated ($n = 6$).
